# Supplementary material for: Nutrient Composition of Autochthonous Beef from Southwest Spain
Source: Foods. 2025 Nov 19;14(22):3961. doi: 10.3390/foods14223961 (PMC12651877; doi:10.3390/foods14223961)
Supplement: Supplementary file 1 [file foods-14-03961-s001.zip › foods-3974709-supplementary.pdf]

Supplementary Table S1. Individual abundance of volatile compound (values are expressed as mean peak area × 10^3) in the *Longissimus dorsi* muscle of five autochthonous cattle breeds from Southwest Spain.

Volatile compund	Breed					SE	*p*-value
	Retinta	Pajuna	Marismeña	Berrenda en Colorado	Lidia		
Acetic acid	221.10^{b}	232.53^{b}	196.57^{b}	223.70^{b}	415.27^{a}	20.17	0.004
Formic acid	2.58^{b}	7.14^{a}	7.62^{a}	7.69^{a}	9.39^{a}	0.44	0.013
Decanoic acid ethylester	2.76^{b}	nd	nd	nd	7.30^{a}	1.39	0.010
Butanoic acid	28.48^{a}	26.12^{a}	7.65^{b}	19.69^{ab}	22.43^{ab}	2.08	0.011
3-Methyl butanoic acid	4.62^{c}	5.29^{bc}	10.56^{a}	5.63^{abc}	9.24^{ab}	0.61	0.003
Hexanoic acid ethyl ester	4.44^{b}	4.32^{b}	4.24^{b}	5.19^{b}	14.26^{a}	1.06	0.002
Acetic acid benzylester	2.92	nd	nd	nd	3.81	0.44	0.349
Pentanoic acid	3.17	3.07	2.49	4.82	3.47	0.29	0.423
Hexanoic acid	17.29^{b}	22.38^{ab}	32.60^{a}	17.01^{ab}	14.27^{b}	1.74	0.029
Hexanedioc acid	3.96	4.03	4.41	4.45	5.25	0.26	0.585
Octanoic acid	6.65^{b}	5.53^{b}	5.79^{b}	5.09^{b}	10.00^{a}	0.43	0.002
Nonanoic acid	7.31	3.95	nd	26.16	8.93	2.56	0.087
Decanoic acid	7.15	5.28	6.42	7.95	9.75	0.51	0.074
Dodecanoic acid	47.76^{a}	13.59^{b}	7.37^{b}	9.28^{b}	61.93^{a}	7.39	0.001
Tetradecanoic acid	9.21^{b}	61.34^{ab}	66.50^{ab}	109.98^{a}	19.98^{b}	9.95	0.004
Hexadecanoic acid	21.33	20.01	47.15	22.37	31.75	3.28	0.111
Octadecanoic acid	23.86	16.17	14.63	22.55	49.46	4.17	0.095
Ethanol	184.69^{b}	257.04^{b}	204.96^{b}	253.99^{ab}	483.34^{a}	29.94	0.010
Butanol	3.72^{b}	5.22^{b}	20.49^{a}	6.90^{b}	4.36^{b}	1.52	0.000
3-Methyl-1-Butanol	3.82^{c}	12.43^{b}	43.98^{a}	11.01^{bc}	12.02^{bc}	2.59	0.000
Pentan-1-ol	19.43	14.37	12.80	13.35	15.25	2.20	0.883
Acetol	4.73	nd	nd	nd	nd	0.70	-
2-Penten-1ol	3.22	nd	nd	nd	nd	0.29	-
Hexan-1-ol	17.17	14.43	40.48	11.60	30.65	3.53	0.098
1-Octen-3-ol	53.15^{b}	57.56^{b}	182.47^{a}	39.34^{b}	79.54^{ab}	13.65	0.020
Heptan-1-ol	6.61	9.05	16.57	7.10	6.89	0.84	0.883
2-Ethyl-1-Hexanol	4.71^{b}	4.06^{b}	7.83^{a}	5.68^{ab}	4.34^{b}	0.81	0.034
2-butoxy ethanol	1.70	12.72	24.70	32.35	4.22	3.71	0.069
1,2-Propanediol	9.52	22.19	22.50	17.59	75.26	10.02	0.279
2,4-decadienol	7.96	5.22	9.43	7.07	8.39	0.66	0.337
Octanol	11.34^{b}	12.76^{b}	20.40^{ab}	43.02^{a}	17.00^{ab}	3.29	0.042
Hexen-3-ol	3.48^{c}	6.98^{b}	9.43^{a}	nd	nd	0.61	0.000
2,3 -Butanediol	33.61^{b}	60.59^{b}	51.32^{b}	54.13^{b}	162.58^{a}	11.67	0.001
2-Decenol	2.91^{b}	3.30^{b}	4.58^{ab}	2.97^{b}	5.54^{a}	0.27	0.001
1,3-Nonadien-3-ol	3.28^{b}	3.69^{b}	6.37^{a}	3.19^{b}	6.35^{a}	0.42	0.028
(Z)-Oct-2-en-1-ol	9.31^{b}	11.94^{b}	27.28^{a}	7.34^{b}	9.55^{b}	1.62	0.003
2-hexadecanol	2.47	3.80	4.18	3.98	4.50	0.22	0.077
2, 7-Octadien-1-ol	4.33^{ab}	3.24^{b}	8.25^{a}	6.52^{ab}	5.48^{ab}	0.55	0.034
4-Decen-1-ol	2.93	3.57	4.97	3.99	2.85	0.27	0.121
(E)-2-Nonen-1-ol	nd	nd	nd	nd	3.28	0.75	-
1-hexadecanol	2.60^{c}	9.04^{a}	4.54^{abc}	7.37^{ab}	2.35^{bc}	0.72	0.000
Benzenemethanol	22.73	3.52	4.83	8.56	4.07	3.35	0.162
Benzeneethanol	3.91^{b}	7.67^{ab}	10.46^{a}	3.90^{ab}	8.90^{a}	0.73	0.003
Ethyl-benzeneethanol	3.63	2.62	4.63	3.59	5.68	0.37	0.066

1-Dodecanol	3.09	2.23	2.78	2.99	2.16	0.25	0.694
Tetradecanol	3.88[c]	12.75[b]	23.12[a]	17.61[ab]	nd	1.81	0.000
2, 7 octadien 1 ol	3.63	3.24	8.25	6.52	7.42	0.63	0.056
Glycerol	nd	3.57	4.97	3.99	nd	1.56	0.105
Phenol	4.04	4.15	6.02	5.50	4.74	0.31	0.294
3-Methyl-Phenol	6.45	4.66	5.96	6.59	7.42	0.36	0.114
2-Methyl-Phenol	5.48	4.10	4.98	8.78	4.31	0.62	0.055
2-Methylbutanal	25.05[b]	22.66[b]	110.29[a]	58.01[b]	27.13[b]	6.99	0.000
Furfural	38.52	27.80	21.71	18.37	28.04	9.12	0.549
Hexanal	261.51	136.26	61.16	83.93	53.39	44.11	0.493
Heptanal	13.24[b]	11.70[b]	44.78[a]	16.63[ab]	19.30[ab]	3.15	0.009
2,4-Nonadienal	11.28[b]	8.08[b]	38.00[a]	7.17[b]	7.17[b]	2.52	0.000
Octanal	5.73	8.21	7.72	5.70	nd	4.85	0.821
Nonanal	75.71	37.04	105.88	61.47	77.23	9.36	0.249
4-Nonenal	4.65	3.63	7.64	5.81	4.59	0.51	0.058
2-Octenal	3.23[ab]	3.66[ab]	1.17[b]	5.55[a]	3.81[ab]	0.47	0.049
Furaldehyde	5.69	nd	nd	nd	7.32	0.69	0.284
Benzaldehyde	100.15	88.48	124.08	91.77	122.82	6.70	0.389
2-Nonenal	10.62	4.03	8.26	11.49	6.74	1.31	0.265
Tridecanal	3.56	nd	nd	nd	nd	0.50	-
(Z)-2Decenal	7.10	nd	nd	nd	4.10	1.18	0.248
Benzeneacetaldehyde	19.26	12.84	23.13	20.87	22.87	1.58	0.212
4-Octadecenal	0.82	3.44	4.67	5.07	8.47	1.15	0.503
Dodecanal	5.74	5.18	3.66	6.49	4.48	0.64	0.832
4-Ethyl-Benzaldehyde	5.56	3.72	3.68	5.70	6.00	0.41	0.173
2-Dodecenal	nd	nd	nd	nd	2.62	1.62	-
(E,E)-2,4-Decadienal	14.97	5.02	4.99	6.04	6.70	1.87	0.235
Tetradecenal	3.20[b]	3.57[b]	5.72[a]	3.60[b]	nd	0.28	0.002
Hexadecanal	27.63[a]	6.86[b]	6.63[b]	6.71[b]	12.36[b]	2.46	0.000
octadecanal	nd	6.13	5.27	10.49	nd	0.98	0.071
Butan-2-one	13.47	nd	nd	nd	13.49	1.04	0.991
2-Heptanone	20.96	10.56	46.65	15.31	2.61	5.52	0.207
Octanal	10.68	4.52	18.10	13.20	27.61	3.55	0.308
Acetoin (3hydroxy-2butanone	33.22	24.60	51.74	25.89	14.69	3.69	0.119
3-Octen-2one	2.54	17.52	14.59	15.97	3.47	4.90	0.352
2-Nonanone	nd	4.46	17.97	3.88	6.42	3.42	0.094
2-Pentadecanona	6.18[a]	2.78[c]	3.22[abc]	2.13[bc]	6.01[ab]	0.47	0.002
3,5-Octadien-2-one	3.81	4.04	5.98	6.84	5.77	0.62	0.094
2-tridecanone	nd	4.11	nd	6.36	3.29	0.80	0.380
Acetophenone	3.25	nd	nd	nd	nd	2.88	-
2-Heptadecanone	nd	4.32	5.66	6.57	7.86	1.08	0.755
Nona-3,5dien-2-one	4.87	nd	nd	nd	nd	1.29	-
2-Octananone	4.60	nd	nd	nd	2.21	0.76	-
3-Nonanone	3.16	nd	nd	nd	nd	0.58	-
Dimethylsulfide	10.99	12.30	13.72	12.83	nd	1.62	0.971
MethylTthiophene	9.62	nd	nd	nd	nd	1.87	-
2-Methyl-Thiazolidine	2.86	nd	nd	nd	3.67	0.40	0.346
Dimethylsulphone	4.33[b]	6.14[b]	3.50[b]	7.30[ab]	16.19[a]	1.25	0.010
Benzyl methyl sulfide	14.10	nd	nd	nd	5.54	2.76	0.180

Benzothiazole	5.53[b]	2.75[c]	5.00[b]	4.95[b]	9.66[a]	0.46	0.000
2,3-DihydroThiophene	9.85	nd	nd	nd	11.98	1.43	0.637
Divinylsufide	6.42[b]	nd	nd	nd	14.63[a]	1.39	0.003
2-Pentyl-furan	18.32	8.56	18.42	7.41	8.09	2.39	0.057
Furfural	4.33	11.26	15.28	17.26	3.28	1.17	-
Acethylfuran	3.55[bc]	2.96[c]	6.73[b]	9.18[a]	4.77[bc]	0.69	0.000
4-Hydroxy-Dihydro2(3H) furanone	14.01	nd	nd	nd	37.62	6.08	0.071
Trimethylamine	16.89	5.49	7.99	4.87	16.29	2.07	0.211
3-Pyrroline	5.73	nd	nd	nd	nd	2.05	-
3-Methyl-Pyrazine	13.13	13.53	41.64	23.11	8.45	3.52	0.069
Pyridine-oxide	2.77[b]	5.17[ab]	5.13[ab]	6.33[a]	nd	0.49	0.038
2,5-Dimethyl-Pyrazine	89.87	35.47	99.15	57.75	16.24	11.93	0.148
2,3,5-Trimethyl-Pyiridne	4.37	4.09	7.00	7.89	2.84	7.93	0.212
2-Ethyl-6-Methyl-Pyrazine	9.93	8.28	5.75	8.07	4.11	2.34	0.109
Trimethyl-Pyrazine	23.38	34.92	nd	26.20	16.81	8.52	0.074
2,6 -Dimethyl-Pyrazine	4.08	nd	nd	nd	nd	0.79	-
2-Ethyl-3, 6, Dimethyl-Pyrazine	nd	11.33	nd	17.65	16.32	8.35	0.436
3,5 Dimethyl-Pyrazole	6.50	10.45	6.58	14.29	9.44	0.92	0.192
Tetramethyl-Pyirazine	3.56	nd	nd	nd	4.87	0.73	0.459
2-methyl-3,5 diethylpyrazine	2.87	5.09	10.11	nd	2.98	1.10	-
Pyrrole	nd	nd	nd	nd	5.93	1.03	-
3-Isobutyl-2,5-Dimethyl-Pyrazine	4.86	nd	nd	nd	nd	0.83	-
2-Ethyl-Pyrrole	8.60	nd	nd	nd	nd	1.82	-
3-Methyl-Butyl-Pyrazine	5.24	nd	nd	nd	nd	0.52	-
2-Isoamyl-6-Methyl-Pyrazine	2.94	nd	nd	nd	3.78	0.34	0.274
2-Butyl-3,5-Dimethyl-Pyrazine	8.53	nd	nd	nd	15.69	1.94	-
3-Isobutyl-2,5-Dimethyl-Pyrazine	9.30	4.78	10.51	8.74	nd	1.22	0.253
2 Acetyl- 3-Methyl-Pyirazine	3.83	3.01	5.34	4.67	nd	0.48	0.455
2 -Allyl-5-Mmethyl-Pyrazine	4.46	nd	nd	nd	nd	1.06	-
Oxime-methoxyphenyl	8.53	7.68	5.67	10.35	9.47	1.01	0.830
2-Acetyl-Thiazoline	5.45	nd	nd	nd	nd	1.07	-
Benzeneacetonitrile	3.14[b]	2.74[b]	3.47[ab]	16.63[a]	nd	1.44	0.021
2-Acetyl-Pyrrole	11.74	8.75	11.91	5.90	4.79	1.13	0.218
2-Pyrrolidinone	5.06	6.48	5.91	8.41	7.24	0.50	0.334
3-Phenylpropionitrile	3.70	3.34	nd	3.86	3.91	0.40	0.958
2,5 Piperazinedione	7.85	nd	nd	nd	8.43	0.92	0.781
1,4 -Dimethyl-piperazine	9.55	6.45	7.35	12.00	7.88	0.65	0.094
Indole	6.76[b]	4.43[b]	5.03[b]	9.59[ab]	20.19[a]	1.44	0.000
Amide	12.41	16.79	14.13	13.78	22.25	1.29	-
Ethyl acetate	nd	nd	nd	nd	10.02	2.86	-
Hexadecanoic acid methylester	nd	4.91	5.86	5.79	nd	0.42	0.059
Heptadecanoic acid ethyl ester	nd	6.78[b]	6.40[b]	10.92[a]	nd	1.22	0.005
4-Ethyl-Benzoic acid 2	2.39	2.94	2.46	5.35	5.50	0.46	0.405

butylester							
Octadecanoic acid methyl ester	13.18[b]	15.11[b]	13.38[b]	46.53[a]	nd	4.17	0.001
Acetic acid methylester 1hydroxi propanone	11.76	4.72	4.77	6.06	6.38	1.06	0.183
Hexadecanoic acid methylester	4.99	nd	nd	nd	7.98	0.91	0.105
Decanoic acid decylester	17.51	15.79	22.59	23.76	26.83	1.55	0.120
Lactic acid ethylester	3.69[b]	3.21[b]	8.19[a]	4.15[ab]	5.79[ab]	0.55	0.021
Toluene	13.03[c]	32.66[b]	30.22[b]	36.03[ab]	47.91[a]	2.84	0.000
Trimethyl-Benzene	nd	13.57	16.13	nd	nd	1.54	0.299
p-Xylene	3.43[b]	6.84[a]	7.80[a]	7.24[a]	5.59[ab]	0.47	0.004
m-Xylene	4.84[b]	5.65[b]	17.63[a]	8.70[b]	6.53[b]	1.34	0.001
Styrene	5.05	6.03	9.16	4.51	4.09	0.62	0.182
m-Cymene	nd	4.31[a]	5.01[a]	3.90[b]	nd	0.20	0.043
phenol 2,6di-terbutyl	3.05	nd	nd	5.01	4.36	0.56	-
1,2,4 Trimethyl-Benzene	3.87	4.38	6.04	9.29	2.85	0.51	0.076
3,5 di-tert-Butyl-phenol	4.63	nd	0.00	nd	nd	1.03	-
Naphthalene	nd	8.37	4.78	6.43	nd	1.68	0.716
2-vinylnaphthalene	nd	2.70	nd	3.65	nd	0.38	0.276
Butyrolactone	12.37[b]	nd	nd	nd	24.35[a]	2.52	0.016
Pentalactone	nd	14.02	6.64	16.99	nd	1.60	0.054
Octalactona	nd	nd	1.65	4.16	nd	2.80	0.062
4-methyldihydro-2(3H)furanone	nd	2.93	2.28	4.35	nd	0.37	0.107
γ-decalactone	nd	3.18[b]	5.77[ab]	6.98[a]	nd	0.66	0.022
Caprolactam	6.94[b]	5.28[b]	4.09[b]	9.75[b]	22.06[a]	1.36	0.000
γ-Octalactone	8.44	2.72	3.98	4.70	4.57	0.90	0.135
